# Central nervous system anomalies in 41 Chinese children incontinentia pigmenti

**DOI:** 10.1186/s12868-024-00872-1

**Published:** 2024-05-21

**Authors:** Li Yin, Zhengyuan Li, Wenjuan Zhan, Yuanjie Kang, Qian Tian, Dan Li, Huifang Zhang

**Affiliations:** 1 Department of Emergency, Xi’an Children’s Hospital (Xi’an Jiaotong University Affiliated Children’s Hospital), No.69, Xiju Yuan Lane, Lianhu District, Xi’an, 86-710003 Shaanxi People’s Republic of China; 2 Department of Imaging, Xi’an Children’s Hospital (Xi’an Jiaotong University Affiliated Children’s Hospital), Xi’an, 86-710003 Shaanxi People’s Republic of China

**Keywords:** Incontinentia pigmenti, Cerebral ischemia, *IKBKG* gene

## Abstract

**Introduction:**

Incontinentia pigmenti (IP) is a rare neuroectodermal dysplasia caused by a defect in the *IKBKG* gene. The pathogenesis of central nervous system injury is believed to be related to microvascular ischemia. Currently, few treatment strategies are available for the inflammatory phase.

**Materials and methods:**

This retrospective descriptive analysis included the clinical data of 41 children with IP collected from 2007 to 2021 in Xi’an, China, comprising clinical characteristics, imaging findings, blood cell analysis, skin histopathology, and genetic data.

**Results:**

Fourteen children (34%) aged 4 days to 5 months exhibited clinical signs and symptoms, including convulsions, delayed psychomotor development following neurological damage, and revealed significant MRI abnormalities, including ischemia, hypoxia, cerebral hypoperfusion, hemorrhage, encephalomalacia, and cerebral atrophy. Eight of the 24 patients (33%) presented with retinal vascular tortuosity and telangiectasis, accompanied by neovascularization and hemorrhage. Thirty-eight children (93%) had elevated eosinophils (mean: 3.63 ± 4.46 × 10^9^), and 28 children (68%) had significantly elevated platelets (mean: 420.16 ± 179.43 × 10^9^). Histopathology of skin revealed microvascular extravasation and vasodilation with perivascular and intravascular eosinophilic infiltration.

**Conclusion:**

Brain injury in IP occurs during infancy until 5 months of age, which is also the acute dermatitis phase accompanied by eosinophilia and an increased platelet count. This study provides evidence of microvascular damage to the skin and fundus during the inflammatory phase. The mechanism of microvascular damage may be similar to that in the brain.

## Introduction

Incontinentia pigmenti (IP) is an X-linked dominant genodermatosis [1]. The disease is caused by a mutation in the nuclear factor kappa B kinase regulatory subunit gamma (*IKBKG*) gene on Xq28 [2, 3], which encodes nuclear factor kappa-B essential modulator (*NEMO*)/inhibitor kappa kinase (IKK)-gamma [4]. It is a rare neuroectodermal dysplasia that affects the skin, hair, teeth, nails, eyes, and central nervous system (CNS) [5]. Skin lesions are typical of this disease and present in the first few weeks of life in almost all affected patients [6]. The incidence of IP is estimated at 0.7 cases per 100,000 births [3]. Characteristic skin lesions evolve through four stages: I. blistering (birth to approximately 4 months of age), II. verrucous rash (for several months), III. swirling macular hyperpigmentation (age ~ 6 months into adulthood), and IV. linear hypopigmentation [2]. Children can have the phases out of the order provided, and do not need to have all the phases. Approximately 30% of patients with IP have neurologic impairments [7]. CNS deficits significantly threaten the normal life of children patients with IP [7]. The frequency of CNS anomalies, similar to the frequency of retinal anomalies [7].These may significantly reduce patients’ quality of life. Neurological involvement is often present along with retinal vasculopathy and may result from ischemia/vaso-occlusive events [2]. However, the mechanisms underlying cerebral and retinal microvascular ischemia are poorly understood, and the lack of appropriate early treatment is a major challenge in the management of IP.

## Methods

### Study design and participants

This was a retrospective, observational study. Clinical data from 41 patients with childhood IP who were referred to the neonatology, neurology, and rehabilitation medicine departments of Xi'an Jiaotong University Affiliated Children's Hospital between January 2007 and December 2022 were collected, in Xi’an, China. There had some overlap between the neonatol participants in this manuscript and our previous article, which was focusing on the characteristics of rash and found eosinophilia and thrombocytosis in the early neonatal period. In this study, we added 9 cases who were older infants or young children and focusing on characteristics of brain injure. The diagnoses were made according to the criteria described by Landy and Donnai [1]. This study was approved by the Institutional Review Board of the Affiliated Children’s Hospital of Xi’an Jiaotong University. Blood samples were obtained from the probands after obtaining written informed parental consent. The clinical features of 41 IP cases were collected from clinical, blood analytical, pathological, radiological, and genetic data. Based on the data from blood cell analysis, skin pathology, fundus examination, and brain imaging, emphasis was placed on the mechanism of cerebral microvascular hypoxia–ischemia in conjunction with the pathogenic mechanism of gene mutation. DNA was extracted from peripheral whole blood samples. *IKBKG* gene analysis was conducted by long-range polymerase chain reaction amplification and Sanger sequencing using a previously reported method [8].

### Statistical analysis

Statistical analysis was performed using SPSS software (version 22.0). Continuous variables were described using the mean, median, and range. Additionally, categorical variables were described as frequency rates and percentages.

## Results

Of the 41 children with IP, four (10%) were male. There were three preterm infants, two of whom were twins, and 38 full-term infants with a mean birth weight of 3037.75 ± 618.99 g. The age at the time of visiting hospital ranged from 1 day to 2 years and 11 months. There were 28 neonatal cases consulted for rash, four of which exhibited neurological signs and symptoms. Ten cases came to our hospital age from 1 month to 2 years and 11 months, because of neurological signs and symptoms. So total 14 cases (34%) had neurological impairment accompanied by clinical signs and symptoms. The remaining three patients were consulted for respiratory infection, diarrhea, and hyperpigmentation. Twenty-four of the 41 patients underwent fundus examination, of which eight (33%) exhibited ocular involvement. All children (100%) presented with skin lesions within one month. Six patients had a family history, and 85% of IP cases were sporadic (Table 1).

**Table 1:** Clinical features and blood cell characteristics of 41 Chinese children incontinentia pigmenti

Patient	Gender	Age at consul-tation	Rash time	Age of present neurological signs	EEG	MRI/CT	Retinal lesions	WBC (× 10^9^)	EO (× 10^9^)	EO%	PLT (× 10^9^)	Genetic testing/family history
1	Female	12 days	At birth	No	Normal	CT: normal	Not done	12	2	16.8	627	Not done/sporadic
2	Female	20 h	At birth	No	Not done	Bilateral white matter decreased symmetrically, with CT value about 17.3–19.3HU	Not done	15.9	2.1	13.4	568	Not done/sporadic
3	Female	4 days	At birth	No	Normal	CT showed symmetrical, low-density, patchy images in the white matter and Subarachnoid hemorrhage	Normal	24.09	7.7	32	285	Not done/sporadic
4	Female	11 days	7 days	No	Normal	CT showed symmetrical, low-density, patchy images in the white matter and subarachnoid hemorrhage, with CT value about 21HU	Normal	26.7	12.2	45.8	474	Not done/sporadic
5	Male	53 days	At birth	Presented at age 50 days with delayed psychomotor development	Not done	CT showed symmetrical, low-density,	Not done	7.16	0.35	4.44	382.4	Not done/sporadic
6	Female	4 days	1 days	Presented at age 3 days with convulsions	Normal	CT showed Hypoxic-ischemic encephalopathy; MRI showed neonatal hypoxic-ischemic encephalopathy with small malacia lesions in the left frontal lobe	Not done	15.2	0.53	3.4	564	Not done/sporadic
7	Female	48 days	1 h	Presented with convulsions at age 36 days and with delayed psychomotor development at age 5 months	Multifocal spike and spike-and-wave discharges	CT showed cerebral ischemia at 20 days; MRI showed brain atrophy at 5 months	Normal	15	1.5	10	694	Not done/sporadic
8	Female	5 days	At birth	No	Normal	CT showed symmetrical, low-density, patchy images in the white matter	Retinal vascular tortuosity and telangiectasis with neovascularization	13.5	2.7	20.2	975	Not done/inherited
9	Female	3 days	At birth	No	Normal	CT showed symmetrical, low-density, patchy images in the white matter	Normal	10.3	0.8	7.4	289	Deletion of exons 4–10 of the IKBKG gene/sporadic
10	Female	12 days	At birth	No	Not done	Not done	Normal	19.7	4.8	24.2	521	Not done/inherited
11	Female	19 days	1 day	No	Not done	MRI normal	Normal	32.2	19.9	61.9	328	Not done/sporadic
12	Female	78 days	At birth	No	Not done	Not done	Normal	18.3	3.17	17.35	547	Not done/sporadic
13	Female	18 h	At birth	No	Not done	MRI showed subarachnoid hemorrhage	Not done	18.8	1.4	7.5	274	Not done/sporadic
14	Female	1 day	At birth	Presented at age 1 day with convulsions	Not done	MRI showed neonatal hypoxic-ischemic encephalopathy in the right frontal lobe, left cerebral hemisphere and right centrum semiovale	Retinal vascular tortuosity and retinal telangiectasis with hemorrhage	15.66	8.02	51.24	139	Not done/sporadic
15	Female	12 h	At birth	No	Not done	MRI normal	Retinal vascular tortuosity and retinal telangiectasis with hemorrhage	5.87	0.66	11.24	163	Deletion of exons 4–10 of the IKBKG gene/sporadic
16	Female	4 days	At birth	No	Not done	Not done	Not done	12.03	1.18	9.74	328	Deletion of exons 4–10 of the IKBKG gene/inherited
17	Female	34 days	At birth	No	Normal	MRI normal	Normal	8.3	0.95	11.44	340	Not done/inherited
18	Female	3 days	At birth	No	Not done	Not done	Not done	23.96	9.54	39.8	563	Deletion of exons 4–10 of the IKBKG gene/inherited
19	Female	3 days	At birth	No	Not done	Not done	Not done	20.7	7.9	37.7	457	Deletion of exons 4–10 of the IKBKG gene Not done/inherited
20	Male	3 days	At birth	No	Not done	Not done	Not done	12.52	3.1	24.4	435	No abnormalities were found
21	Female	24 days	28 days	No	Not done	Not done	Not done	15.5	3.3	19	420	Heterozygous mutation c.519-3_519dupCAGG
22	Female	8 days	At birth	No	Normal	MRI normal	Normal	18.81	6.1	32.5	553	Deletion of exons 4–10 of the IKBKG gene
23	Female	5 months	At birth	Presented at age 4 months with convulsions	Not available	CT showed cerebral ischemia at age 23 days; MRI showed left periventricular leukomalacia with decreased regional perfusion at 5 months	Not done	13.72	3.69	26.9	598	Not done/sporadic
24	Female	5 months	1 day	No	During waking, the right side of the brain discharges asynchronously	MRI showed right periventricular leukomalacia and small amount of subdural effusion on the forehead	Normal	10.66	0.95	8.9	338	Not done/sporadic
25	Female	2 months	At birth	No	Slightly more early discharge during sleep	MRI showed Multiple acute and subacute cerebral infarction at 2 months	Not done	15.6	1.87	12	389	Not done/sporadic
26	Female	11 days	At birth	Presented at age 11 days with convulsions	Not done	MRI normal	Familial exudative vitreoretinopathy-like changes with no V-shaped demarcations or peripheral neovascularization	28.43	14.93	52.5	403	Not done/sporadic
27	Female	5 days	At birth	No	Multifocal spikes, sharp waves	MRI showed neonatal hypoxic-ischemic encephalopathy and subdural hemorrhage of the tentorium	Normal	9.08	2.42	22.2	398	Deletion of exons 4–10 of the IKBKG gene
28	Female	3 days	At birth	No	Not done	MRI normal	Normal	10.54	2.15	20.4	335	Not done/sporadic
29	Female	3 days	At birth	No	Normal	MRI normal	Retinal vascular tortuosity and retinal telangiectasis with right retinal hemorrhage	12.72	1.27	11	203	Deletion of exons 4–10 of the IKBKG gene
30	Female	4 days	At birth	No	Not done	Not done	Not done	13.69	1.63	11.9	254	Deletion of exons 4–10 of the IKBKG gene
31	Female	5 days	At birth	No	Normal	MRI showed neonatal hypoxic-ischemic encephalopathy and Suboccipital subdural hemorrhage	Retinal vascular tortuosity and retinal telangiectasis with hemorrhage	14.18	3.31	23.4	580	Not done/sporadic
32	Female	1 days	At birth	No	Not done	Ultrasound: pairs of side ventricles of the brain white matter enhanced echo	Normal	8.38	0.61	7.3	182	Not done/sporadic
33	Female	1 days	At birth	No	Multifocal spikes, sharp waves	MRI normal	Normal	12.79	2.78	21.8	354	Deletion of exons 4–10 of the IKBKG gene
34	Female	42 days	At birth	At age 15 days presented with convulsions	Not done	MRI showed neonatal hypoxic-ischemia at age 5 days; softening and atrophy in the left frontoparietal region at age 42 days	Retinal hemorrhage and vascularization of omentum vicryl was not seen in the peripheral region	30.32	11.3	37.2		Not done/sporadic
35	Female	2 years and 11 months	Not know	No	Not done	MRI showed cerebellar tonsillar hypoplasia with enlarged foramen median and occipital	Reduction of the optic disc	8.13	0.76	9.3		Not done/sporadic
36	Female	6 months	At birth	No	Not done	Not done	Not done	18.35	0.2	0.5	832	Not done/inherited
37	Female	3 days	At birth	No	Multifocal spikes and sharp waves	MRI normal	Not done	4.22	0.9	7.4	356	Deletion of exons 4–10 of the IKBKG gene
38	Female	5 months	Not know	No	Normal	MRI showed that the bilateral ventricles, prepontine cistern, and cisterna magna were enlarged	Not done	5.55	0.04	0.7	126	Deletion of exons 4–10 of the IKBKG gene/sporadic
39	Female	19 days	At birth	No	Multifocal spikes and sharp waves	MRI showed white matter damage in the left area of cerebellar vermis and the right frontal cortex and subarachnoid hemorrhage	Normal	9.3	0.73	7.8	389	Not done/inherited
40	Male	12 days	3 days	No	Not done	MRI normal	Normal	11.42	0.79	6.9	510	Not done/sporadic
41	Male	8 months	30 days	5 days	Hypsarrhythmia, particularly in sleep stage	MRI showed leukomalacia in the periventricular region and multiple malacia foci in the bilateral thalamus and cerebellum	Not done	8.58	1.38	16.1	305	Not done/sporadic
Mean								15.32 ± 7.27 × 10^9^	mean: 3.63 ± 4.46 × 10^9^	19.30 ± 15.03%	420.16 ± 179.43 × 10^9^	

*MRI* cranial magnetic resonance imaging, *CT* cranial computerized tomography, *EEG* electroencephalogram, *WBC* white blood cell, *PLT* platelet, *EO* eosinophil, *EO%* eosinophil percentage

### Central nervous system manifestations

Of the 41 children, 24 underwent cranial magnetic resonance imaging (MRI) examination (two underwent cranial computerized tomography (CT) at the time of initial diagnosis and cranial MRI at follow-up), nine underwent cranial CT, and one underwent cranial ultrasound (Table 1).

**Fig. 1:**
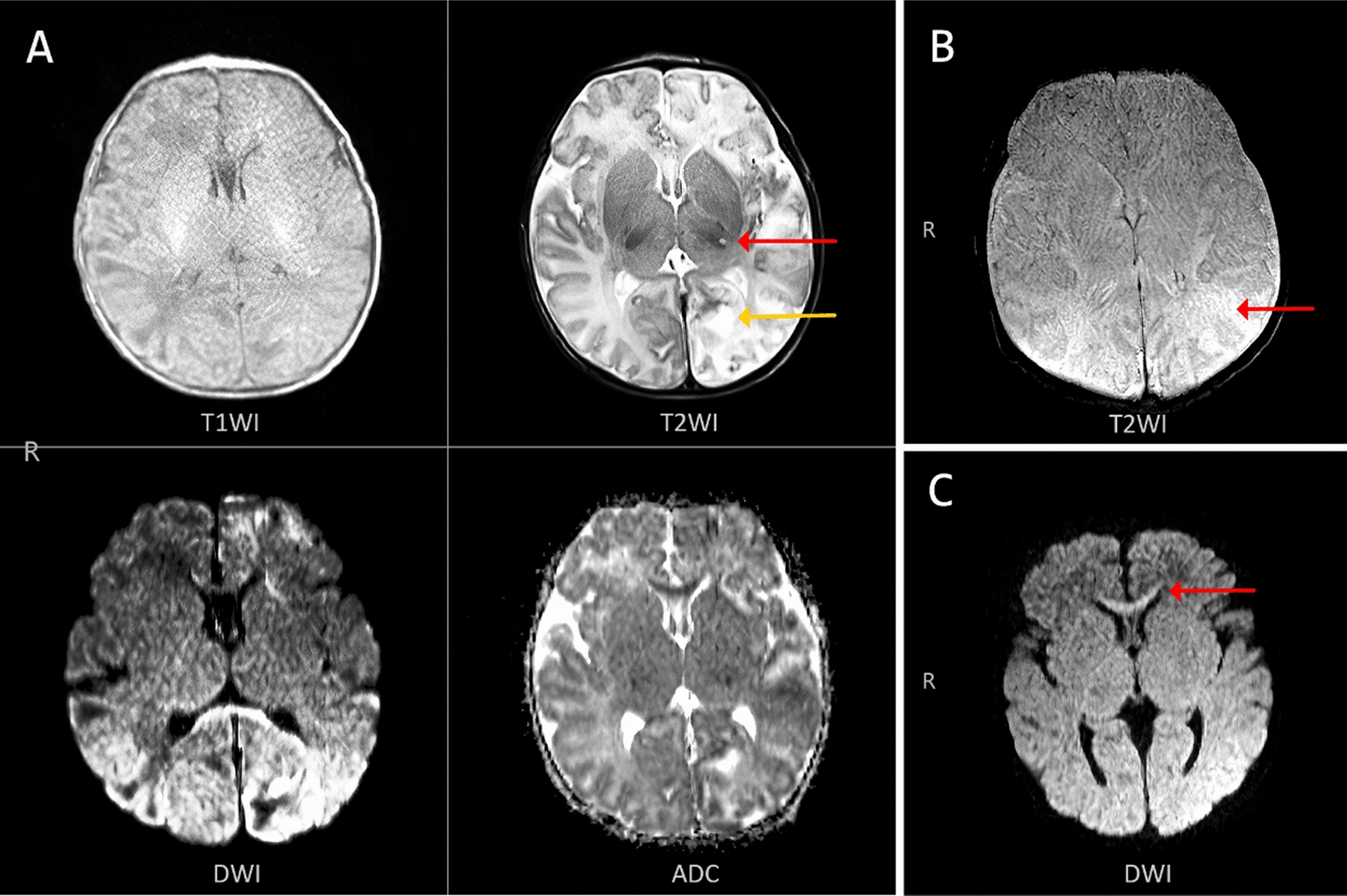
MRI of brain ischemia and hemorrhage: **A** The MRI of the 14th patient showed T1- and T2-weighted extensive signal intensity changes. Diffusion Weighted Imaging(DWI) and Apparent Diffusion Coefficient(ADC) showed high-signal-intensity lesions in the left cerebral hemisphere and right frontal lobe, which were proposed to be hypoxic infarction. **B** The MRI of the 31th patient showed occipital subdural hemorrhage. **C** The MRI of the 14th patient showed genu of the corpus callosum depicting high signal intensity

**Fig. 2:**
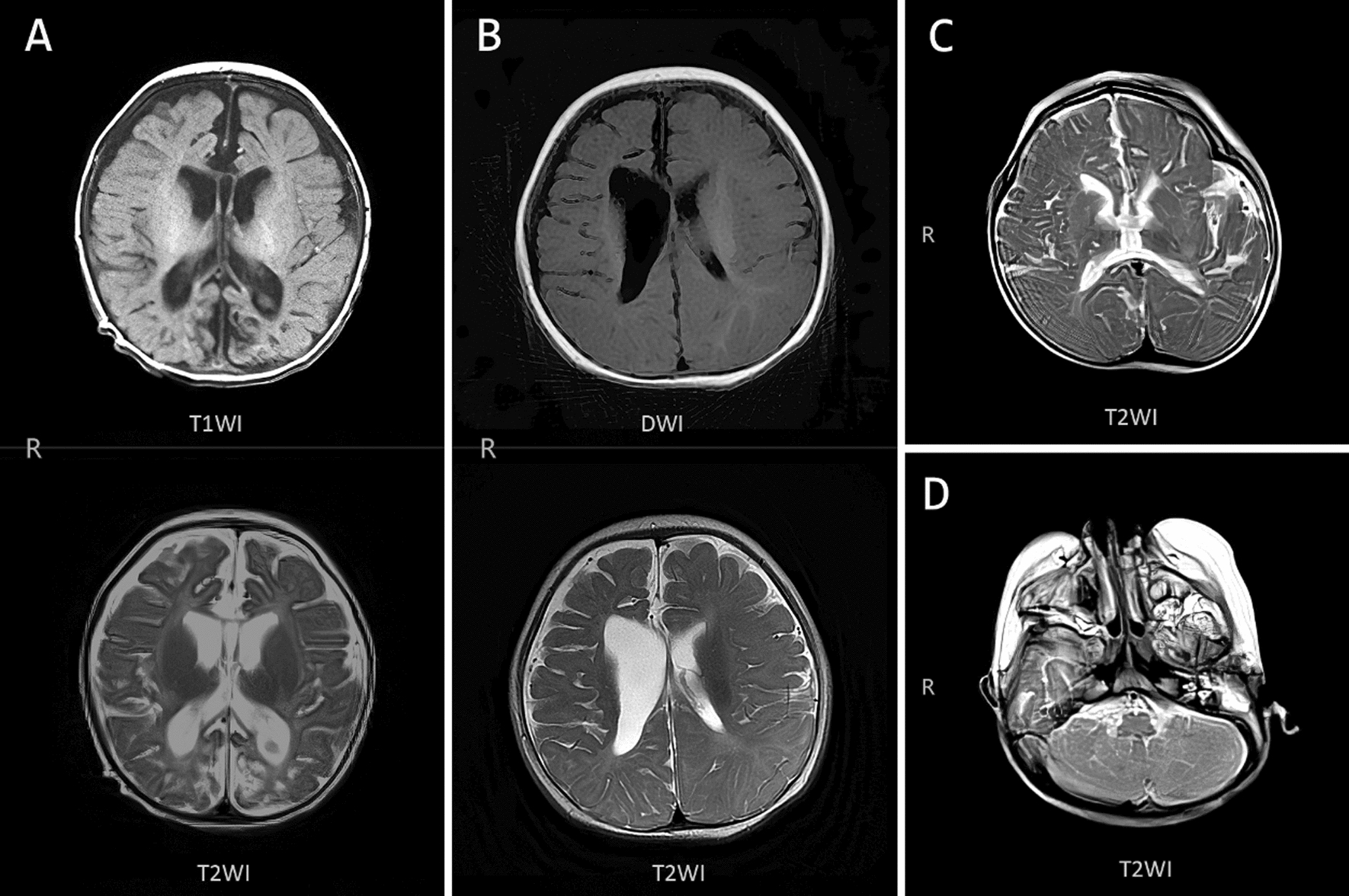
MRI of brain atrophy and encephalomalacia: **A** The MRI of the 7th patient showed T1- and T2-weighted MRI showing brain atrophy. **B** The MRI of the 24th patient showed T1- and T2-weighted MRI showing right periventricular leukomalacia. **C** The MRI of the 41th patient showed T2-weighted MRI showed encephalomalacia at the thalamus. **D** The MRI of the 41th patient showed T2-weighted MRI showing cerebellar encephalomalacia

Of the 24 cases examined by cranial MRI, 14 (34%) presented with significant abnormalities on cranial MRI such as ischemia, hypoxia, cerebral hypoperfusion (Fig. 1A), and hemorrhage (Fig. 1B) during the neonatal period and brain atrophy (Fig. 2A) and encephalomalacia (Fig. 2B) during infancy. Abnormal signal intensities were also relatively common in the corpus callosum (Fig. 1C), cerebellum (Fig. 1A, yellow arrow; Fig. 2D), basal ganglia, internal capsule, and thalamus (Fig. 1, red arrow; Fig. 2C). The ages of the 14 children presenting with neurological signs and symptoms ranged between 4 days and 5 months. With follow up the 14 children (34%) with many MRI changes and early seizures who show seizures and delayed psychomotor development. According follow up, the other 10 cases underwent cranial MRI, 9 cases underwent cranial CT, and one underwent cranial ultrasound had no obvious imaging abnormalities in the early stage who show normal intelligence.

All nine cases of cranial CT were reported to have ischemic-hypoxic encephalopathy, primarily hypointense white matter, with some cases of small amounts of subarachnoid hemorrhage. One case with convulsion of the nine cranial CT cases underwent cranial MRI re-examination after 1 week indicated small encephalomalacic lesions in the left temporal lobe. Another case of the nine cranial CT cases showed delayed psychomotor development at the 5-month follow-up, and cranial MRI re-examination indicated cerebral atrophy. In one case by cranial B-scan ultrasonography indicated bilateral lateral ventricular white matter hyperechogenicity.

Seven of the 41 cases had convulsions. The time of onset of convulsions ranged from the day of birth to 4 months of age; there was only one case without any abnormalities on cranial MRI, and the remaining six cases exhibited abnormalities on cranial MRI. Nineteen patients underwent electroencephalogram examination, of which eight exhibited abnormalities, including multifocal, multifocal spikes, and spike-and-wave discharges. One patient who experienced frequent convulsions exhibited a high degree of dysrhythmia during sleep.

### Ocular manifestations

**Fig. 3:**
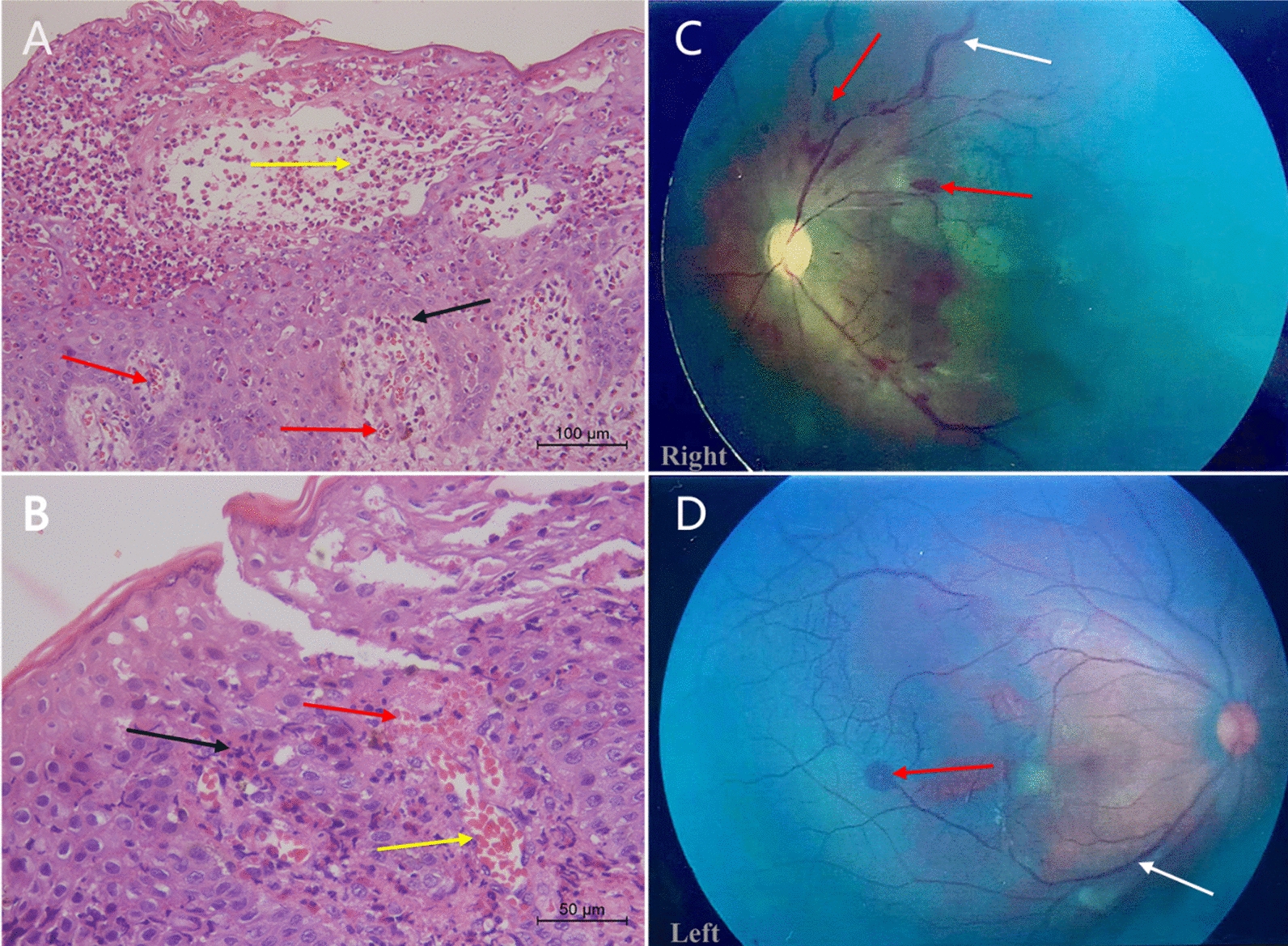
Histopathologic characteristics and fundus photographs. **A** Spongiotic dermatitis with eosinophilia in vesicular stage eosinophils in blister cavity (yellow arrow). Densely distributed eosinophils in the periphery (black arrow) and significant infiltration of eosinophils into the microvasculature (red arrow). (Hematoxylin and eosin stain; original magnification × 200). **B** The microvasculature was dilated and extravasated (yellow arrow) and appeared to form thrombi (red arrow). Densely distributed eosinophils in the periphery of microvessels (black arrow) (Hematoxylin and eosin stain; original magnification × 400). **C** and **D** Retinal vascular tortuosity and retinal telangiectasis (white arrow), with hemorrhage (red arrow), avascular areas and neovascularization

Twenty-four of the 41 children underwent fundus examination. There were eight cases (33%) of retinopathy. All eight cases presented with retinal vascular tortuosity and retinal telangiectasis (Fig. 3C, D white arrow); two cases presented with neovascularization, five cases with retinal hemorrhage (Fig. 3C, D, red arrow), and one case with familial exudative vitreoretinopathy-like changes with no V-shaped demarcations or peripheral neovascularization. At follow up, there was one case of retinal detachment and one of bilateral optic disc reduction. Four of eight children with fundus damage also presented with brain damage (Table 1).

### Histopathologic characteristics

All patients underwent skin biopsy for pathological examination and made diagnosis. The biopsy material sampled during the vesicular stage exhibited spongiotic dermatitis with massive intraepidermal and dermal eosinophilia (Fig. 3A) and eosinophil-filled intraepidermal vesicles (Fig. 3A, yellow arrow). The microvasculature was dilated and extravasated (Fig. 3B, yellow arrow), and appeared to form thrombi (Fig. 3B, red arrow) with densely distributed eosinophils in the periphery (Fig. 3A, B, black arrow) and significant infiltration of eosinophils into the microvasculature (Fig. 3A, red arrow).

### Laboratory findings

Blood cell analysis revealed that 32 of the 41 children (78%) had significantly elevated white blood cells (WBC) (range: 5.55–34.3 × 10^9^, mean:15.32 ± 7.27 × 10^9^), 38 cases (93%) had elevated eosinophils (range of eosinophil count: 0.04–19.9 × 10^9^, mean: 3.63 ± 4.46 × 10^9^; range of eosinophil percentage: 0.50–61.9%, mean: 19.30 ± 15.03%), and 28 cases (68%) had significantly elevated platelets (range: 126–975 × 10^9^, mean: 420.16 ± 179.43 × 10^9^) (Table 1). Forty children had normal high-sensitivity C-reactive protein (hs-CRP) (< 0.5 mg/L), and there was one case with concomitant Crohn’s disease where hs-CRP was 188 mg/L. The normal range of WBC is 5.0–12.0 × 10^9^. The normal range of eosinophil count is 0.05–0.5 × 10^9^. The normal range of eosinophil percentage is 0.5–5.0%. The normal range of platelets count is 125–350 × 10^9^.

### Characteristics of dermatologic lesion

**Fig. 4:**
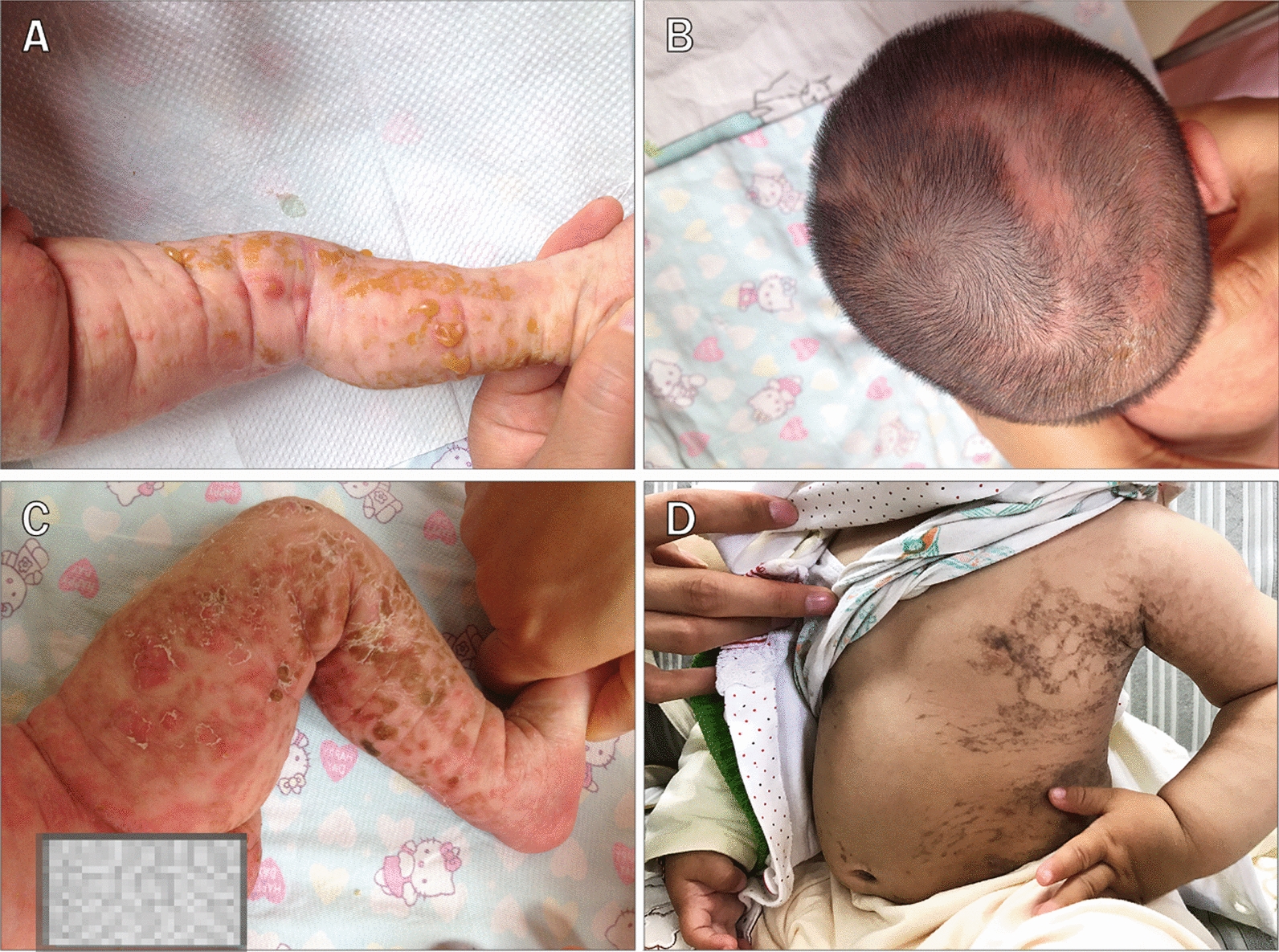
Dermatologic lesions: **A** Erythema and superficial vesicles on the inflammatory bases with a linear distribution presented since birth. **B** Linear vertex alopecia associated with scarring presented since birth. **C** verrucous papules with red macule. **D** reticulated hyperpigmentation. The consent from the patients' parents for publication of the patients' photographs was obtained

Dermatological findings are typically the first signs of IP. Twenty-nine patients (71%) were diagnosed in the neonatal period upon being consulted for a rash, and the remaining patients presented with a rash at early stages that was not accurately identified or diagnosed, and was instead diagnosed as impetigo, syphilis, or an unknown diagnosis. Thirty patients (73%) showed erythema at birth, indicating that they had lesions in utero. Thirty-five patients (85%) at the vesicular stage exhibited characteristic dermatological lesions during the first weeks of life. The vesicular stage (stage I) was characterized by erythema and superficial vesicles on the inflammatory bases with a linear distribution (Fig. 4A). Erythema and superficial vesicles were usually absent on the face, although scalp lesions were relatively common (Fig. 4B). The blisters ranged in diameter from 1 mm to 1 cm or greater. The cutaneous manifestations developed into new erythema with new linear vesicles erupting, and older lesions developed into verrucous papules (stage II, Fig. 4C), followed by hyperpigmentation (stage III, Fig. 4D). Follow-up indicated that dermatitis was most severe during the first month after birth, regressed slowly after 4 months, and eventually disappeared by 1 year of age.

### Variant in NEMO

Genetic testing was performed in 14 children, of whom 12 (79%) carried a deletion of exons 4–10 of the *IKBKG* gene. One case carried microdeletions of the *IKBKG* gene, a heterozygous mutation c.519-3_519dupCAGG, but the heterozygous mutation was not found in the baby’s mother. Only one male patient underwent genetic testing, and no abnormalities were found.

## Discussion

Of the 41 patients, the mean weight of the full-term infants was 3037.75 ± 618.99 g, indicating that IP does not significantly impair growth and development during pregnancy. IP is typically lethal in males during embryogenesis [9]; however, four patients (10%) in the present study were male. Only one male patient underwent genetic testing, and no abnormalities were found. This can be explained by somatic mosaicism, hypomorphic mutations, or the presence of an extra X chromosome, as in Klinefelter syndrome [2].

Thirty-one children presented with abnormalities on cranial imaging, with neurological signs and symptoms in 14 cases (34%), all of whom eventually underwent cranial MRI. MRI findings were suggestive of hypoxic-ischemic changes accompanied by hemorrhage. Hennel et al. reported a case of a neonate with IP and seizures on day 4 of life who underwent MRI and angiography scanning at age 8, 13, and 21 days. The serial magnetic resonance images demonstrated the evolution of acute microvascular hemorrhagic infarcts in the periventricular white matter in the first week of life. Magnetic resonance angiogram revealed decreased branching and poor filling of the intracerebral vessels [10]. Thus, the pathophysiological mechanism of brain damage in IP is reduced cerebral perfusion, which in turn leads to vascular occlusion and hemorrhage and causes hypoxic-ischemic changes in the brain.

The clinical observations in the present study showed that more severe skin damage was associated with higher WBC and eosinophil and platelet counts. Dilated and extravasated skin capillaries with perivascular and intravascular eosinophil infiltration were observed upon histopathological examination of the skin samples (Fig. 3A and B). Stalled leukocytes in and around capillaries can also release lysosomal enzymes—including various protein and lipid hydrolases such as cathepsins and collagenases—and oxygen radicals, which can break down tissue proteins, damage the vascular endothelium, and destroy collagen and fibronectin, thus causing structural damage to the vessel wall and hemorrhage. Retinal vascular tortuosity and telangiectasis with retinal hemorrhage were observed in the fundus examination samples (Fig. 3C, D), supporting this hypothesis. This hemorrhage, in turn, stimulates hemostatic platelet aggregation, resulting in microthrombus formation (Fig. 3B, red arrow), leading to hypoxic ischemia and apoptosis of the cells in the supplied area. Neurons in the brain cannot regenerate after apoptosis, which leads to brain atrophy and delayed psychomotor development after brain injury.

Fundus vasculature changes in IP, including vascular tortuosity, hemorrhage, presence of avascular areas, and neovascularization (Fig. 3C, D), were consistent with occlusive and ischemic vascular changes and supported our hypothetical mechanism of cerebral ischemia [11]. Skin damage is observed in all patients with IP and is generally most evident shortly after birth, up to four months of age [1]. Fourteen patients exhibiting abnormalities on MRI presented with signs and symptoms of neurological injury within the first 5 months of life. Thus, we hypothesized that brain injury in IP occurs during infancy, which is also the acute phase of dermatitis, consistent with our rationale for brain injury due to microvascular pathology.

Mutations in the *IKBKG* (formerly known as *NEMO/IKKγ*) gene located at Xq28 have been found to cause the disease expression [4, 6]. The most common genetic mutation in IP is an approximately 11.7-kb deletion in the *IKBKG* gene that removes exons 4 through 10. This mutation accounts for 70–80% of patients with IP worldwide [12, 13]. The present study reported 11 children (79%) carrying deletions in exons 4–10 of *IKBKG* and two cases of microdeletions in the *IKBKG*. One male was not tested for any abnormalities. The low-level mosaicism that occurs in male patients may escape molecular investigation if the methodology is used in relation to female patients [14, 15].

*IKBKG*, a 48-kd protein, is an essential component of the newly discovered nuclear factor κB (NF-κB) signaling pathway [16]. When activated, NF-κB controls the expression of multiple genes, including cytokines and chemokines, and protects cells against apoptosis. The mechanism by which *IKBKG* deficiency via the NF-κB pathway causes the phenotypical expression of the disease has recently been elucidated [6]. In IP, the *IKBKG* derangement results in a truncated NF-κB that either is unable to protect against apoptosis or becomes pro-apoptotic; hence, cell death can occur in response to a variety of potential stimuli [11, 17]. In the present study, more severe dermatitis was associated with a higher WBC count, predominantly eosinophils, in the peripheral blood. Eosinophil recruitment occurs via the release of eotaxin by activated keratinocytes [10]. Recruited eosinophils undergo degranulation and release proteases, leading to inflammation in the epidermis and other areas of the body [6, 18]. In the event where NF-κB-deficient endothelial and other cells throughout the body have overexpression of chemotactic factors such as eotaxin, specific to eosinophils, may result in systemic eosinophilia [19, 20]. The presence of eosinophils, in combination with other inflammatory factors, leads to extensive inflammation. Endothelial inflammation results in vaso-occlusion and ischemia, which contribute to retinal and neurological manifestations. Occlusion of the retinal arteries leads to areas of avascularity and underperfusion, precipitating ischemia. Neovascularization occurs as a sequela to this [21, 22]. In the CNS, brain atrophy and other neurological sequelae are thought to share a similar vaso-occlusive ischemic pathophysiology with retinal ischemic events [4, 7]. The mechanism of elevated platelet levels remains unclear, but is considered to be associated with severe inflammatory responses, microvascular endothelial cell injury, and microvascular bleeding. Elevated platelet levels increase the likelihood of microthrombus formation and blockage of cerebral blood vessels.

Thus, we recommend the following to prevent and treat early brain injury in patients with IP: (1) Inhibition of the inflammatory response. In children with severe inflammation, glucocorticoid treatment is recommended to inhibit inflammation [23]. T. I. Kaya reported therapeutic use of topical corticosteroids in the vesiculobullous lesions was effectived [24]. It is hope to reduce brain and fundus injury by oral and IV steroids. Recently, anti- tumor necrosis factor had been used for the purpose with success [4]. Additionally, the skin should be protected against bacterial infections. Retinoids have been reported to regress painful verrucous tumours [23]. (2) Oral dipyridamole should be used to inhibit platelet aggregation, reduce thrombus formation, and prevent occlusion of cerebral blood vessels. (3) Recent studies have reported excellent anatomical outcomes years after laser photocoagulation of the ischemic retina. Early data indicate that antivascular endothelial growth factor therapy can induce retinal revascularization but runs the risk of late recurrent neovascularization and requires long-term monitoring [25]. The clinical presentation of IP varies widely from mild skin damage to severe brain damage and retinal detachment. Varying degrees of disease severity require different levels of multidisciplinary involvement, including neonatology, dermatology, ophthalmology, neurology, and dentistry. Therefore, multidisciplinary treatment, management, and follow-up of patients with IP are essential. Routine blood cell analysis, cranial MRI, fundus examination, EEG, skin biopsy, and genetic testing are recommended for disease confirmation during the dermatitis phase soon after birth. Fundus examination and cranial MRI are recommended on a monthly basis until 4 months of age, and then every 6 months until the dermatitis phase subsides. The patients may be then followed-up by ophthalmologic and neurological physicians.

## Conclusion

Central nervous system and fundus damage pose a serious threat to the normal life expectancy and quality of life of patients with IP. Brain injury occurs in patients with IP during infancy, which is also an acute phase of dermatitis. Retinal vascular tortuosity, telangiectasis, hemorrhage, skin microvascular extravasation, and vasodilation with perivascular and intravascular eosinophil infiltration may be consistent with occlusive and ischemic vascular changes in cerebral ischemia. Few treatment strategies are currently available for the inflammatory phase of IP. The present study suggests that based on the early inflammatory response and the mechanism of brain injury, early glucocorticoid treatment to suppress severe inflammatory responses and dipyridamole treatment to inhibit platelet aggregation may be effective; however, this must be confirmed during clinical treatment.

## Data Availability

The datasets generated and analyzed during the current study are not publicly available for ethical reasons, but are available from the corresponding author upon reasonable request.
